# Enhancing the biomethane production from lignocellulosic residues through bioaugmentation of anaerobic digestion

**DOI:** 10.1007/s00449-025-03223-4

**Published:** 2025-08-18

**Authors:** Jamie K. D. van Wyk, Daneal C. S. Rorke, Johann F. Gӧrgens, Eugéne van Rensburg

**Affiliations:** Department of Chemical Engineering, Stellenbosch University, Private Bag X1, Matieland, Stellenbosch, 7602 South Africa

**Keywords:** Anaerobic co-digestion, Cellulolytic bacteria, Corn stover, Alkali pretreatment, Facultative anaerobic microorganisms

## Abstract

Bioaugmentation of anaerobic digestion (AD) systems is considered a cost-effective and environmentally friendly strategy to combat incomplete digestion of recalcitrant lignocellulosic substrates. This study investigated the lowest microbial inoculum size required for once-off bioaugmentation of AD cultures to enhance biomethane yield and process performance. The batch, laboratory-scale anaerobic co-digestion was carried out using pretreated corn stover (PCS) and food waste (FW), with cellulolytic *Bacillus subtilis, Serratia marcescens* and* Bacillus licheniformis.* The bioaugmentation screening was accomplished through a stepwise increase in the microbial loading using an initial standardised concentration of 0.4 × 10^11^ colony-forming units (CFU)/mL within the system. Bioaugmentation decreased the digestion time by up to 11 days. The inoculation of *B. subtilis* at a microbial concentration of 20 × 10^11^ CFU/mL (4.85 g DCW/L) improved the biomethane yield by 34% compared to the unaugmented control and produced 525 NmL CH_4_/gVS. Additionally, *S. marcescens* at 12 × 10^11^ CFU/mL doubled the volumetric methane productivity from 0.47 ± 0.02 to 1.04 ± 0.02 mL/(mL.day) when compared to the unaugmented control. The application of Nanopore sequencing after AD, to investigate the microbial community dynamics and structure in this treatment, underlined 43.52, 7.69 and 25.26% increases in the bacterial alpha diversity, namely the Shannon-, Simpson- and Observed indices, respectively. Moreover, a high abundance of between 50 and 80% of the *Firmicutes* population was identified.

**Supplementary Information:**

The online version contains supplementary material available at 10.1007/s00449-025-03223-4.

## Introduction

Across the globe, many countries remain heavily dependent on fossil-based energy systems, despite their environmental, social, and economic disadvantages [1]. In turn, agricultural residues remain largely underutilised, offering minimal economic value [2, 3]. Through the conversion of bio-based resources such as lignocelluloses into energy, a transition from fossil fuels to renewable energy together with a circular waste economy and improved resource management can be achieved [4]. A transition towards renewable technologies further promotes the use of lignocellulosic material as an organic resource with inherently high energy potential [5]. Lignocelluloses such as corn stover (CS), derived from *Zea mays L*. as one of the most abundant agricultural crops, are characterised by a high carbohydrate content in the leaves, shells, tassels and cobs. After the harvest season, large amounts of corn stover remain for animal feed or more often are disposed of through incineration [6].

Anaerobic digestion (AD) for biogas (methane) production is an established renewable energy technology that allows for environmentally friendly, efficient conversion of organic materials using a microbial consortium in an oxygen-free environment, which can support the circular economy [4, 7]. Based on the chemical composition of CS, a theoretical methane yield between 431.2 and 564 mL-CH4/gVS has been proposed [8, 9]. However, as lignocellulosic conversion during AD is mostly limited by the hydrolysis stage, pretreatment of CS prior to AD is preferred to unlock this energy potential [3]. The interconnected nature of cellulose, lignin and hemicellulose in lignocelluloses forms a rigid physicochemical structure which is recalcitrant to biological degradation in the AD process. Consequently, the difficult and slow hydrolysis of lignocellulosic biomass such as wheat straw, CS and sugarcane bagasse, limits the biomethane yields to 55–67% of the theoretical maximum [8, 10].

An appropriate pretreatment technique can disrupt the structural composition of lignocellulose, increasing the accessibility to cellulose and hemicellulose carbohydrates [5]. The reduction of lignin, a non-carbohydrate component, is often one of the objectives of pretreatment due to the limited number of microorganisms that can degrade this complex component under anaerobic conditions [11]. While alkaline, acidic, thermal, and mechanical pretreatment processes have been employed in AD, alkaline pretreatment has several benefits including a low severity of pretreatment, focus on delignification, limited production of inhibitory compounds that might negatively affect the microbial consortium and high efficiency [12–14].

Beyond the aforementioned benefits of pretreatment, bioaugmentation can also improve the AD of lignocelluloses like CS. Bioaugmentation entails the supplementation of microorganisms into the AD microbial community to enhance the performance of the system, specifically targeting rate-limiting steps [15]. As AD is typically used as a wastewater treatment system, the innate microorganisms found in AD are not sufficiently exposed to lignocelluloses and therefore exhibit less efficient lignocellulose degradation mechanisms [16]. Microbial bioaugmentation with function-specific microbiota is thus suggested as a cost-effective, time-efficient and more robust alternative to enzyme supplementation to improve hydrolysis of lignocelluloses in AD [12, 15, 17]. Table 1 highlights bioaugmentation studies carried out in AD systems using monocultured strains. Interestingly, augmentation of the AD of brewery spent grain with *R. flavefaciens* resulted in a 5.6% reduction in methane production when compared to its unaugmented control, while *P. xylanivorans* augmentation of AD under the same conditions improved methane yield by 17.8%. This observation demonstrates the strain-specific sensitivity of augmenting microorganisms to the innate AD microbial community, with many augmenting microorganisms not surviving long enough to impact the process.

**Table 1:** An overview of single-strain bioaugmentations of AD systems to enhance methane production

Substrate	Pretreatment	Bioaugmentation in AD	AD conditions	AD outcome	Refs
Corn stover	Microaerobic, with *B. subtilis* 5 mL O_2_/gVS, 24 h	–	37 °C; ISR = 1:2; pH = 7.2; HRT = 57 d	270.8 mL CH_4_/gVS (+ 17.4%) Control: unaugmented, untreated AD)	[18]
Birchwood chips	Steam explosion; 210 °C, 10 min	*Caldicellulosiruptor bescii* (2% dose)	62 °C; ISR = 2:1; pH = 7.5; HRT = 50 d	197 mL CH_4_/gVS (+ 142.1%) Control: untreated, unaugmented AD)	[19]
Wheat straw (WS) co-fed with cow manure (CM)	–	*Clostridium thermocellum* (3.3% v/v per day)	53 °C; OLR = 1.6 gVS/L; CM:WS = 9:1	396 mL CH_4_/gVS (+ 15.8%) Control: unaugmented co-AD)	[20]
WS co-fed with CM	–	*Melibacter roseus* (3.3% v/v per day)	53 °C; OLR = 1.5 gVS/L; CM:WS = 9:1	368 mL CH_4_/gVS (+ 7.6%) Control: unaugmented co-AD	[20]
Brewery spent grain	–	*Ruminococcus flavefaciens* 007C (5% v/v)	37 °C; HRT = 30 d;	209.41 mL CH_4_/gVS (-5.6%) Control: unaugmented AD	[21]
Brewery spent grain	–	*Pseudobutyrivibrio xylanivorans* Mz5^T^ (5% v/v)	37 °C; HRT = 30 d;	261.31 mL CH_4_/gVS (+ 17.8%) Control: unaugmented AD	[21]
WS	–	*Clostridium cellulolyticum* (25 mL/g WS)	37 °C; ISR = 2:1; HRT = 35 d	342.5 mL CH_4_/gVS (+ 13%) Control: unaugmented AD	[22]

*ISR* inoculum-to-substrate ratio; *HRT* hydraulic retention time; *OLR* organic loading rate

A very high increase in methane yield of 142.1% was reported by Mulat et al. [19] due to the augmentation of AD of steam-exploded birchwood chips with *C. bescii*. However, 118% of this improvement was credited to the application of steam explosion as a pretreatment, illustrating the role that increased access to hydrolysable organics plays in efficient AD systems. Angelidaki & Ahring [23] compared bioaugmentation with a hemicellulolytic bacterium to supplementation with (hemi)cellulolytic enzymes during the AD of cow manure fibre. Whereas enzyme supplementation resulted in minimal improvements, a 30% improvement in the biomethane yield was achieved using microbial bioaugmentation. However, these bench-scale (approximately 30 mL working volume) results were obtained by incubating the fibres before AD in a concentrated culture of the augmenting microorganism.

Bioaugmentation with facultative anaerobic microorganisms is a feasible option for bioaugmentation in AD [11, 24, 25]. However, significant modification to the AD process is often required to see significant improvements. Nzila et al. [25] discuss a 15-day feedstock pretreatment by Zhong et al. [26] with an anaerobic consortium of *Saccharomyces cerevisiae, Coccidioides immitis, Hansenula anomala, B. licheniformis, Pseudomonas sp., B. subtilis, Pleurotus florida* and *Lactobacillus deiliehii.* This bioaugmentation improved methane production by 76%: however, although successful, the inclusion of such a lengthy pretreatment before AD significantly reduces plant productivity. Facultative anaerobes of the *Bacillus, Serratia* and *Meliobacter* genera have been shown to exhibit promising benefits such as increased secretion of cellulase activity (0.44–0.75 U/mL) [27] and enhancements in biomethane yields from AD [20, 28, 29]. The facultative anaerobic microorganisms also provide the benefit of functioning under both anaerobic conditions and aerobic respiration, thereby avoiding potential AD process instabilities associated with the inability of obligate anaerobic, cellulolytic microorganisms to survive when exposed to limited oxygen amounts in these systems, as seen with *Clostridium sp.* bioaugmentation [11, 22, 30].

Important considerations for the application of bioaugmentation in AD of lignocellulosic biomass have been reported, such as the selection of microorganisms that can effectively enhance yields [25, 31, 32], effective microbial loading concentrations [33, 34], cultivation conditions [35], and the consequential effects on the indigenous microbial community [36–39]. However, microbial bioaugmentation is mostly limited to facultative anaerobic microorganisms, such as from the *Bacillus* genus. It is typically applied in aerobic pretreatments or for the preparation of crude enzyme products [40–42], with substantial variations in the effects of bioaugmentation on AD systems. Bioaugmentation with facultative anaerobes under anaerobic conditions will result in a substantially different metabolic state to aerobic conditions [43–45], which may assist with the AD of lignocellulosic biomass for biomethane production [46–48]. The *Bacillus* genus consists of a broad range of strains with cellulolytic activities in bioaugmentation [29, 43, 49, 50], which may affect microbial augmentation AD systems in different ways. However, there is a paucity of previous reports on the impacts of practically feasible microbial loadings or dosages of single-strain hydrolytic cultures used for bioaugmentation of one-stage batch AD systems [33, 34, 51], limiting the capacity to determine strain-specific actions on an AD microbial community.

The present study assessed the effect of bioaugmentation with three alternative facultative anaerobic, cellulolytic species namely, *Bacillus subtilis, Bacillus licheniformis* and *Serratia marcescens* on biomethane production from pretreated CS (PCS). The microbial loadings of the preferred bacterial species, added as monocultures to AD systems, were optimised to maximise methane production during co-digestion of PCS with food waste (FW) in bench-scale anaerobic digesters. Nanopore sequencing technology was employed to assess changes in the microbial consortium after augmentation with a selected treatment, while the compositional changes of the lignocellulosic content in the feedstock were monitored and used to evaluate the enhancement in digestion or hydrolysis in addition to measuring the biomethane production performance.

## Materials and methods

### Feedstock preparation

Air-dried CS bales were obtained from local farms in the Western and Eastern Cape, South Africa after a late harvest season. The CS was knife-milled to a reduced particle size of 6 mm and then sieved to remove dust particles using an automated sieve. Thereafter, it was subsampled using the cone and quarter method before storage at room temperature. Prior to use in AD, the CS was subjected to alkali pretreatment by soaking the CS in 15% ammonium hydroxide loaded into a sealed glass jar at a 1:6 (w/w) CS-to-NH_4_ ratio and incubated for 12 h at 60 °C in a temperature-regulated water bath, as described by Kim & Lee [52]. The pretreated substrate was then washed with deionised water until a neutral pH was reached. The solid fraction was utilised as the pretreated corn stover (PCS) in the feedstock analysis and AD assessments.

The mixed food waste (FW) used as a co-substrate was collected from a local grocery store and consisted of a defined ratio of various spoiled fruit, vegetables, and food. The FW was homogenised using a bowl cutter and blender, aliquoted and stored at −20 °C until use. Digested cow manure as an inoculum source was obtained from an anaerobic digestor located on a dairy farm in Darling, South Africa. It was degassed at mesophilic conditions (37 °C) in a 30-L continuous stirred-tank reactor (CSTR) for one week before use. As the digestate was sourced over a period of 24 months, the potential effect of seasonal variability on the digestate was taken into consideration.

### Bioaugmentation strains

#### Microbial cultivation and enumeration

*Bacillus subtilis* BD170*, Bacillus licheniformis* ATCC 14580 and an unclassified *Serratia marcescens* strain were provided by the Department of Microbiology at Stellenbosch University. The streaked strains were pre-cultured (17 h; 37 °C; 120 rpm) in nutrient broth containing (in g/L) peptone, 15; yeast extract, 3; sodium chloride, 6; and glucose, 1 (Sigma Aldrich®) before their use in the subsequent bioaugmentation AD experimental work. Additionally, 40% (v/v) glycerol stocks were made with pre-cultured microorganisms for general storage at −80 °C.

The enumeration of the respective microbial strains to determine the concentration as CFU/mL for bioaugmentation was conducted using the plate-counting method. Only individual plates with 25–250 colonies each were considered in the enumeration. The corresponding dry weight was determined using the gravimetric oven method at 60 °C. The absorbance at 600 nm was measured every two hours over a period of 24 h to create growth curves of the three strains.

#### Microbial enzyme activity determination

The maximum microbial enzyme production (U/mL) was determined according to the procedure described by Arju Hossain et al. [17]. After centrifugation at 4660 rpm for 10 min, the resultant supernatant of the culture broth was used as the crude enzyme. Exoglucanase activity was assessed using 50 mg filter paper (Whatman 1; 1 cm × 6 cm) and endoglucanase activity was assessed using 1 mL of 1% CMC-Na dissolved in 0.05 M citrate buffer. Deionised water replaced the crude enzyme solution in the negative control.

1 $${\text{Enzymatic}}\,{\text{activity}}\left( {\frac{{\text{U}}}{{{\text{ml}}}}} \right) = \frac{{{\text{Reducing}}\,{\text{sugar}}\,{\text{concentration}}\,\, \times \,1000\,\, \times \,{\text{dilution}}\,{\text{factor}}}}{{{\text{Glucose}}\,{\text{molecular}}\,{\text{weight}}\, \times \,{\text{incubation}}\,{\text{time}}\,({\text{minute}})}}$$

### Experimental procedure

The biomethane potential (BMP) assays were performed in triplicate using the Automated Methane Potential Test System and Gas Endeavour® (BPC instruments). The experiments were conducted using 600 mL Schott bottles loaded to a working volume of 400 mL under mesophilic conditions controlled at 37 °C. The PCS and FW were used at a C:N ratio of 30:1 and the TS content of the substrate was standardised to 10% (w/w) by dilution with deionised water. Furthermore, all treatments were tested at an inoculum-to-substrate ratio (ISR) of 2:1 (based on the volatile solids (VS) content) [53]. Once cell growth reached an OD_600_ of 1.0–1.1, the cells of the augmenting microorganisms were harvested by centrifugation at 4660 rpm for 10 min. Before inoculation into the reactors, the harvested cells were resuspended in phosphate buffered saline (PBS) at an inoculation volume of 10% (v/v) to eliminate the influence of additional growth media in the AD system. A standardised bacterial cell count of 0.4 × 10^11^ CFU/mL served as a baseline for inoculation dosages, and each incremental increase was a multiple of the baseline cell count. An upper limit was set to 20 × 10^11^ CFU/mL due to the culture volume (up to 6 L per 400 mL assay) required to obtain these cell concentrations. The inoculation ratios per treatment feedstock and bioaugmentation seed are described in Table 2, with each treatment denoted with a key description. Throughout the operating period of each BMP assay, a triplicate reactor set containing inoculum with water was used and denoted as the ‘blank’ for calculation purposes. Additionally, a set of positive controls containing inoculum with Avicel® microcrystalline cellulose also formed a part of the assays for quality control of the system. The individual reactors were flushed with a 40:60 nitrogen-carbon dioxide gas mixture for ± 1 min to establish an anaerobic environment before the software system was started. The digestion period lasted until the daily biomethane production fell below 1% of the cumulative biomethane production [53].

**Table 2:** Bioaugmentation treatment assays, with the corresponding loadings used (microbial loading, substrate loading and inoculum loading) per litre of AD volume

		Microbial loading			Inoculum loading		Substrate loading	
	Treatment	CFU/mL (× 10^11^)	DCW (g/L)	% of TS added	DW (g/L)	% of TS added	DW (g/L)	% of TS added
*B. licheniformis*	BL-1	0.4 ± 9.2 × 10^8^	0.017 ± 0.001	0.04	29.35	73.79	10.41	26.17
	BL-2	0.8	0.034	0.08	29.54	71.62	11.67	28.30
	BL-3	1.2	0.050	0.12	29.54	71.59	11.67	28.29
	BL-5	2.0	0.084	0.18	32.63	70.42	13.63	29.40
	BL-10	4.0	0.168	0.27	43.40	70.36	18.12	29.37
	BL-30	12	0.504	0.81	43.40	69.98	18.12	29.21
	BL-50	20	0.840	1.39	42.22	70.04	17.22	28.57
*B. subtilis*	BS-1	0.4 ± 26 × 10^8^	0.097 ± 0.006	0.24	29.35	73.64	10.41	26.11
	BS-2	0.8	0.195	0.47	29.54	71.34	11.67	28.19
	BS-3	1.2	0.292	0.70	29.54	71.17	11.67	28.13
	BS-5	2.0	0.487	1.04	32.63	69.81	13.63	29.15
	BS-10	4.0	0.973	1.56	43.40	69.45	18.12	28.99
	BS-30	12	2.919	4.53	43.40	67.36	18.12	28.11
	BS-50	20	4.850	7.54	42.22	65.67	17.22	26.79
*S. marcescens*	SM-1	0.4 ± 14 × 10^8^	0.016 ± 0.001	0.04	29.35	73.79	10.41	26.17
	SM-2	0.8	0.032	0.08	29.54	71.62	11.67	28.30
	SM-3	1.2	0.048	0.12	29.54	71.59	11.67	28.29
	SM-5	2.0	0.080	0.17	32.63	70.42	13.63	29.40
	SM-10	4.0	0.160	0.26	43.40	70.37	18.12	29.22
	SM-30	12	0.480	0.77	43.40	70.01	18.12	29.22
	SM-50	20	0.800	1.33	42.22	70.08	17.12	28.59

*CFU* Colony-forming units; *DCW* Dry cell weight; *TS* Total solid; *DW* Dry weight

### Analytical methods

#### Feedstock analysis

The TS and VS were measured gravimetrically using a standard procedure [54]. The TS was determined by drying the samples at 105 °C for 24 h and VS was measured by igniting the succeeding TS samples at 550 °C for 2 h. Carbon, hydrogen, nitrogen, and sulphur concentrations (%) were determined by the Central Analytical Facility (CAF) in Stellenbosch, using the Elemental Vario EL cube Analyzer (protocols no. ASTM D4239 and ASTM D5373). Lignocellulose fibre determination was conducted using NREL-LAP procedures according to Sluiter et al. [54]. Acid detergent fibre (ADF), neutral detergent fibre (NDF), and crude fibre contents of lignocellulose-containing samples were determined by NviroTek Labs, Wellington, South Africa. The pH was measured using a calibrated pH probe (Hanna® Instruments). Volatile fatty acids (VFAs) and sugar concentrations were determined using High-Performance Liquid Chromatography (HPLC). The VFA content was tested in filtered samples in the pH range of 3–7 using a BioRad HPX-87H column (250 × 7.8 mm with a guard cartridge) at conditions of 65 °C and 210 nm UV wavelength.

#### Productivity analysis

The system’s overall efficiency in producing biomethane is demonstrated by the volumetric methane productivity rate (VMPR) assessment, which uses the system’s production volume and technical time to produce 80% of the volume as parameters [55].

2 $$VMPR = {{V1} \mathord{\left/ {\vphantom {{V1} {(V2)(T80)}}} \right. \kern-0pt} {(V2)(T80)}}$$

V1 = final cumulative methane amount (mL) according to the digestion period.

V2 = The working volume of the reactor (mL).

T_80_ = The shortest time (days) required to achieve 80% of V1.

#### Microbial community analysis

Quick-DNA Faecal/Soil Microbe Miniprep extraction kits (Zymo Research) were used per the recommended protocol to isolate the total genomic DNA from the samples obtained from BMP runs. To prepare for the Nanopore sequencing, the region-specific primers (27F 5’-AGAGTTTGATCCTGGCTCAG-3’ and U1492R 5’-GGTTACCTTGTTACGACTT-3’) were used for the unidirectional sample multiplexing at the 16S rRNA V1-V9 bacterial region. The 25 μL reaction setup contained 1.5 mM MgCl2, 0.5 μL dNTP mix (10 mM solution), 1 × Hotstart Buffer (KAPA Taq ™), 0.5 U Hotstart DNA Polymerase (KAPA Taq ™), 0.25 μM forward and reverse primers and 1.5 μL of template DNA [56]. The targeted regions were extracted through PCR amplification by an initial process of denaturation at 95 °C for 5 min, followed by 35 cycles at 95 °C for 30 s each, an additional cycle at 56 °C for 30 s, a cycle at 72 °C for 30 s. Lastly, a final extension was carried out at 72 °C for 30 s [56]. The resultant PCR concentrations were analysed using a BIODROP spectrophotometer before being subjected to flow cells in the Oxford Nanopore Technologies® MinION™ Mk1B device.

The Mothur software (v1.48.0) was used to analyse the FASTQ files [57]. Branch-point sequences (bps) that were between the lengths of 1000–1800 bps, had clear bases, scored a mean quality score of ≥ 20, and contained homopolymer regions lengthened at < 8 base pairs were used for further analysis. The SILVA v138.1 (http://www.arb silva.de/) database was used for the sequence classification. Graphs were created using the Mothur (v1.48.0) software and the Microeco package in R (v4.2.2) [58]. Additionally, the alpha diversity indices (Shannon index, Simpson index and Observed OTUs) were assessed to provide insight into the richness, evenness and diversity within the communities.

#### Statistical analysis

One-way ANOVA and LSD post-hoc tests at a 95% confidence level were used for the determination of statistically significant biomethane yield results. Values were expressed as mean ± standard deviation. Statistically significant differences were considered when *p* < 0.05.

## Results and discussion

### Feedstock characterisation

The chemical compositions of the food waste (FW), untreated corn stover (UCS) and pretreated corn stover (PCS) are shown in Table 3. The carbon and nitrogen content of the UCS of 43.38 and 0.78%, respectively, corresponding to a C:N ratio of 55.97, aligned with the carbon content of 41.4% and C:N ratio of 51.75 reported by Ajayi-Banji et al. [59]. However, effective conversion of the available carbohydrates of the UCS (Table 3) for biomethane production requires an appropriate pretreatment to reduce its recalcitrance to microbial and enzymatic degradation [60]. Pretreatment using ammonium hydroxide increased the hemicellulose and cellulose contents by means of selective removal of lignin, decreasing the lignin content of CS from 23 to 14% (Table 3). Such delignification of lignocelluloses is known to increase susceptibility to microbial and enzymatic degradation [61], such as microbial strains used for bioaugmentation. The high C:N ratio of the pretreated substrate of 67.74 highlighted the need for nitrogen supplementation. Food waste with a nitrogen content and C:N ratio of 1.67 and 28.62, respectively, was utilised as the co-substrate to improve the balance between carbon and nitrogen (Table 3). The food waste was mixed with the PCS to achieve a resultant C:N ratio of 30:1, which was used for subsequent experiments, and was within the recommended C:N ratio range of 20:1–30:1 for bioavailable feedstock to be digested during a stable AD process [62].

**Table 3:** Characterisation of the substrates used in the present study

Parameters	Food waste	Untreated corn stover	Pretreated corn stover
Carbon (%TS)	51.13	43.38	43.36
Nitrogen (%TS)	1.67	0.78	0.64
Hydrogen (%TS)	16.76	6.62	7.10
Sulphur (%TS)	bdl	bdl	bdl
C:N ratio	28.62	55.97	67.74
*Total solids (% w/w)	18.37 ± 0.29	92.45 ± 0.12	13.51 ± 0.46
*Volatile solids (% w/w TS)	17.48 ± 0.21	86.99 ± 1.27	97.26 ± 3.18
*Cellulose (% TS)	–	22.20 ± 0.17	24.97 ± 0.02
*Hemicellulose (% TS)	–	14.30 ± 0.14	16.23 ± 0.15
*Lignin (% TS)	–	22.82 ± 0.05	14.13 ± 0.08

*Values are expressed as mean ± standard deviation (*n* = 3); *bdl* below detection level (< 0.04% for Sulphur)

### Bioaugmentation strains characterisation

**Fig. 1:**
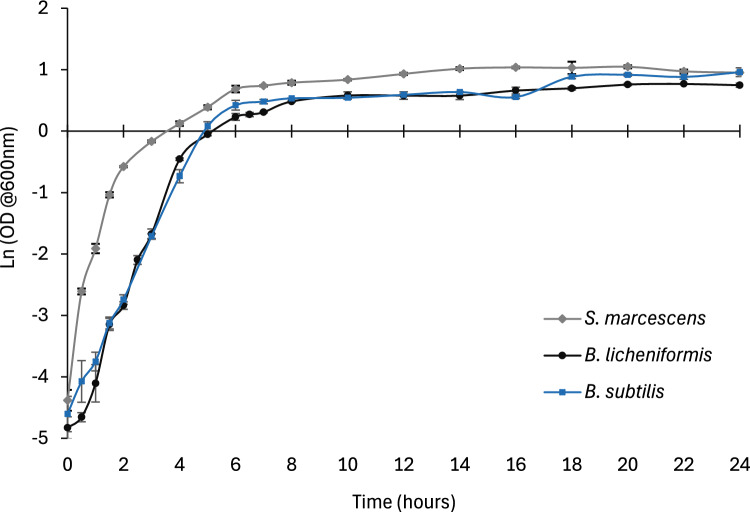
The natural logarithmic cell growth curve of three bioaugmentation strains cultivated over a period of 24 h at 37 °C and 120 rpm in nutrient broth at a pH of 7. *S. marcescens* indicated by diamond, *B. licheniformis* indicated by circle, and *B. subtilis* indicated by square coordinates

Key growth and enzyme production parameters by the selected *B. subtilis, B. licheniformis* and *S. marcescens* strains are shown in Table 4. Growth curves (Fig. 1) showed the late exponential phase to be around the 6-h growth point for all three strains; this was the established time point for cell harvest due to maximal biomass concentration (0.4 × 10^11^– 40 × 10^11^ CFU/mL) retrievable at this stage (Table 4). Among the three strains, *S. marcescens* displayed the most efficient growth, illustrated by a faster maximum specific growth rate (*μ*_max_; 1.33 h^−1^) and higher biomass concentration (2.8 × 10^12^ CFU/mL), compared to *B. subtilis* (1.00 h^−1^) and *B. licheniformis* (1.19 h^−1^). *S. marcescens* had a much higher growth rate compared to Kurniawan et al. [63]’s research (0.256 h^−1^) using TSY media, but a closer result (0.91–1.11 h^−1^) to *S. marcescens* ATCC 13880 and *Serratia* sp. HRI isolates in assessments using Luria broth with or without different disinfectants [64]. Likewise, *μ*_max_ values obtained for the two *Bacillus* strains were lower than the results obtained by Vehapi et al. [65] for *Bacillus subtilis* ATCC 6633 in Tryptic Soy media at optimised conditions (2.42 h^−1^), but were within the range of 0.79 h^−1^–1.1 h^−1^ reported by Berbert-Molina et al. [66] for the fermentation tests on *Bacillus thuringiensis* var. *israelensis* IPS 82 using Luria broth with varying glucose concentrations (10–152 g/L).

**Table 4:** Growth and enzyme production parameters for *B. subtilis, B. licheniformis* and *S. marcescens* at the time of harvest after a 6-h cultivation period

Parameter	*B. subtilis*	*B. licheniformis*	*S. marcescens*
Maximum specific growth rate,* µ*_*max*_ (h^−1^)	1.00 ± 0.01	1.19 ± 0.04	1.33 ± 0.07
Biomass concentration (CFU/mL)	4.0 × 10^10^ ± 2.6 × 10^9^	1.4 × 10^11^ ± 6.2 × 10^9^	2.8 × 10^12^ ± 7.4 × 10^10^
Exoglucanase activity (U/mL)	0.022 ± 0.003	0.022 ± 0.004	0.015 ± 0.000
Endoglucanase activity (U/mL)	0.040 ± 0.003	0.035 ± 0.003	0.035 ± 0.002

The secretion of cellulases and cellulolytic characteristics of the strains selected for bioaugmentation were confirmed by enzymatic assays of the cultivation supernatant. After assessment of both endoglucanase and exoglucanase enzyme production, all three strains exhibited similar final enzyme concentrations to each other, in the range of 0.015–0.040 U/mL, while the higher biomass concentrations of *S. marcescens* did not correlate with higher enzyme activity in comparison to the *Bacillus* strains (Table 4). The measured enzyme activities were similar to those reported by Deka et al. [67] and Shyaula et al. [68], but substantially lower than the 0.44–0.75 U/mL reported for some *Bacillus* genera [29, 69]. The low enzyme activities further warrant the use of concentrated microbial cultures for augmentation.

### Impact of bioaugmentation on AD process performance

**Fig. 2:**
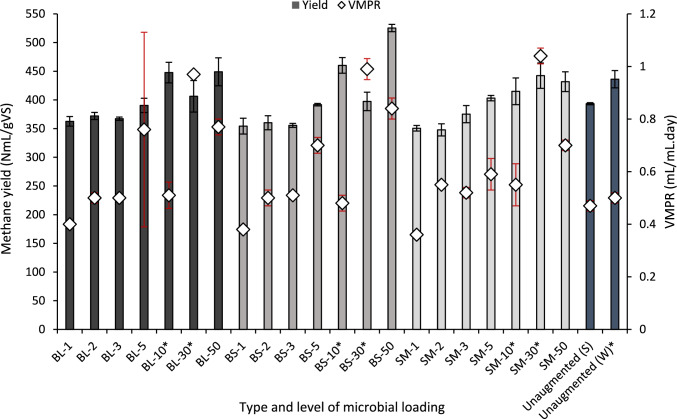
The biomethane yield (NmL/gVS) and volumetric productivity rate (VMPR) (mL/mL.day) of the bioaugmented AD processes at varied inoculum concentrations, compared to their respective unaugmented controls. *BL* *Bacillus licheniformis*, *BS* *Bacillus subtilis*, *SM* *Serratia marcescens.* The un-augmented AD results obtained during the winter (W) season are comparable to microbial loadings with a signified (*), whereas the un-augmented summer (S) season is comparable to the rest of the microbial loadings tested. Standard deviation of *n* = 3

**Fig. 3:**
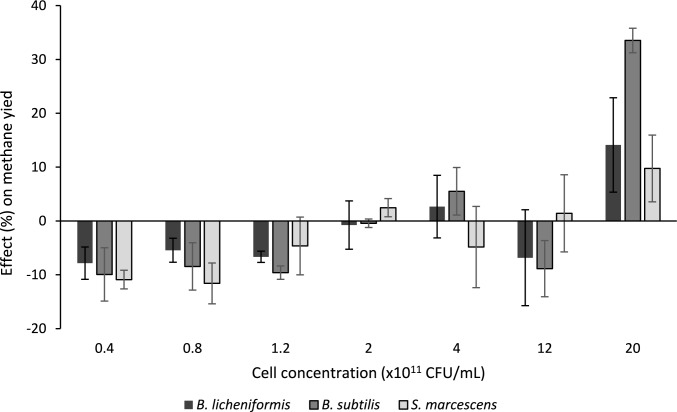
The enhancing or reducing effect (%) of bioaugmentation treatments in AD, using the three pure strains inoculated at various concentrations (CFU/mL) within the reactors, when compared to their respective, unaugmented controls

Figure 2 displays the effect of the bioaugmentations on the AD process in terms of methane yield and VMPR, compared to the respective unaugmented controls. Figure 3 illustrates the enhancement or reduction (%) in the methane yields after assessment of the various bioaugmentation treatments, in comparison to the unaugmented treatment.

#### Role of strain selection and inoculum size on biomethane yield

The BMP assessment indicated that a minimum inoculum size of 20 × 10^11^ CFU/mL (after supplementation of AD culture with bioaugmentation strain) was required to substantially increase biomethane yields, as observed for bioaugmentation treatments BS-50, BL-50 and SM-50 (Fig. 3). The highest biomethane yield of 525.35 mL/gVS (*p* < 0.05) was recorded for *B. subtilis* using an inoculum size of 20 × 10^11^ CFU/mL, which represented a 34% improvement in the biomethane yield compared to the control. Augmentations with *B. licheniformis* and *S. marcescens*, respectively, produced a small but significant (*p* < 0.05) margin of improvement in the methane yield of 14 and 10%, respectively, when compared to their unaugmented control (Fig. 3).

It is generally expected that increasing the inoculum size of the bioaugmentation culture will provide a proportional improvement in AD performance [33]. This would be evident in methane yields and survival of the bioaugmentation culture, especially with repeated inoculation throughout the digestion process [25, 33, 70, 71]. However, in the current study, the bioaugmentation inoculation was only done once, at the beginning of the AD process, which may necessitate a larger inoculum compared to the repeated inoculations previously reported. A one-time dose, or too small a dose results in a reduced microbial population of the augmenting strain, in comparison to the innate microbial community. Once exposed to the complex environmental conditions of an AD system, augmenting strains (a) interact with the innate microorganisms found in AD systems and (b) may not portray their typical behaviour seen in lab cultures, affecting their likelihood of survival. This can have a positive or negative effect on the microbial balance within the system, which can have a negative or positive effect on methane yield [72].

Figure 3 shows that incremental increases from 0.4 × 10^11^ to 12 × 10^11^ CFU/mL of the once-off bioaugmentation did not result in a corresponding increase in methane yield, as there was no significant increase in the yield below the threshold inoculum loading of 20 × 10^11^ CFU/mL. Additionally, the three lowest inoculum sizes investigated decreased the biomethane yield (Fig. 3), potentially due to population dynamics in the AD microbial consortium, where the bioaugmentation strain may either encourage or inhibit other strains based on its abundance in the system [38]. This effect was significant (*p* < 0.05) when *B. subtilis* was used at the three lowest inoculum loadings, as well as when *S. marcescens* was used at the two lowest inoculum loadings. Liu et al. [73] describe the competitive action between augmented *B. subtili*s and native *Bacillus* species in the AD system, which results in a general reduction in both populations. Liu et al. [73]’s lowest *B. subtilis* dose, which was equivalent to the highest dose in the current study was most significantly reduced over time. This led to the survival of other strains during the hydrogenotrophic stage, which indirectly affected methane production.

Obi et al. [74] describes the hydrolytic and acidogenic capacity of *S. marcescens* 39_H1 to enhance the hydrolysis of lignocellulose in an AD system: however, if the strain has difficulty surviving, the pathogenic nature of *S. marcescens* may contribute to reduced methane yields by secreting secondary metabolites and antimicrobial peptides, which may have a negative impact on the innate AD microorganisms [75]. Evaluation of a range of bioaugmentation microbial loadings allows determination of the ratio between augmenting and indigenous strains that create synergistic balance, although raising the bioaugmentation microbial dosage beyond the concentration of 20 × 10^11^ CFU/mL was deemed impractical due to the large volume of cultivation material required.

#### Improvements in productivity and solids degradation of the AD process from bioaugmentation

Bioaugmentation enhanced the volumetric productivity rate of all the AD processes, except the lowest of the microbial loadings tested (Fig. 2). By reducing the total AD process time by 2–11 days, bioaugmentation increased the VMPR of the process, demonstrating improved efficiency in generating methane [55]. For example, a methane yield of 442.32 ± 22.09 NmL/gVS was obtained by the SM-30 bioaugmentation in 25 days compared to 436.12 ± 15.09 NmL/gVS obtained by its unaugmented control in 27 days; exhibiting an increase in VMPR of 108%. Although the *S. marcescens* SM-30 bioaugmentation provided the largest-observed increase in VMPR, this was not associated with the largest reduction in its digestion time, but the speed at which 80% of the methane was produced. The discrepancy highlights that, despite the increased substrate availability through faster hydrolysis, the limiting step in the process was shifted towards the other phases of the process, such as methanogenesis [76]. The positive effect of bioaugmentation on an AD system’s VMPR has been reported for other augmenting strains [20, 77–79]. Although specific VMPRs were not provided, Linsong et al. [34] reported a reduction of 3–20 days in the time required to achieve 80% of the methane yields (T_80_), with increased bioaugmentation loadings corresponding to greater reductions in T_80_. Given the prevalent concern of prolonged digestion or hydrolysis time required for the AD of lignocellulosic substrates, the implementation of bioaugmentation provided the expected benefits of increasing the rate of substrate degradation, which is typically highlighted as the rate-limiting step in the process [12].

**Fig. 4:**
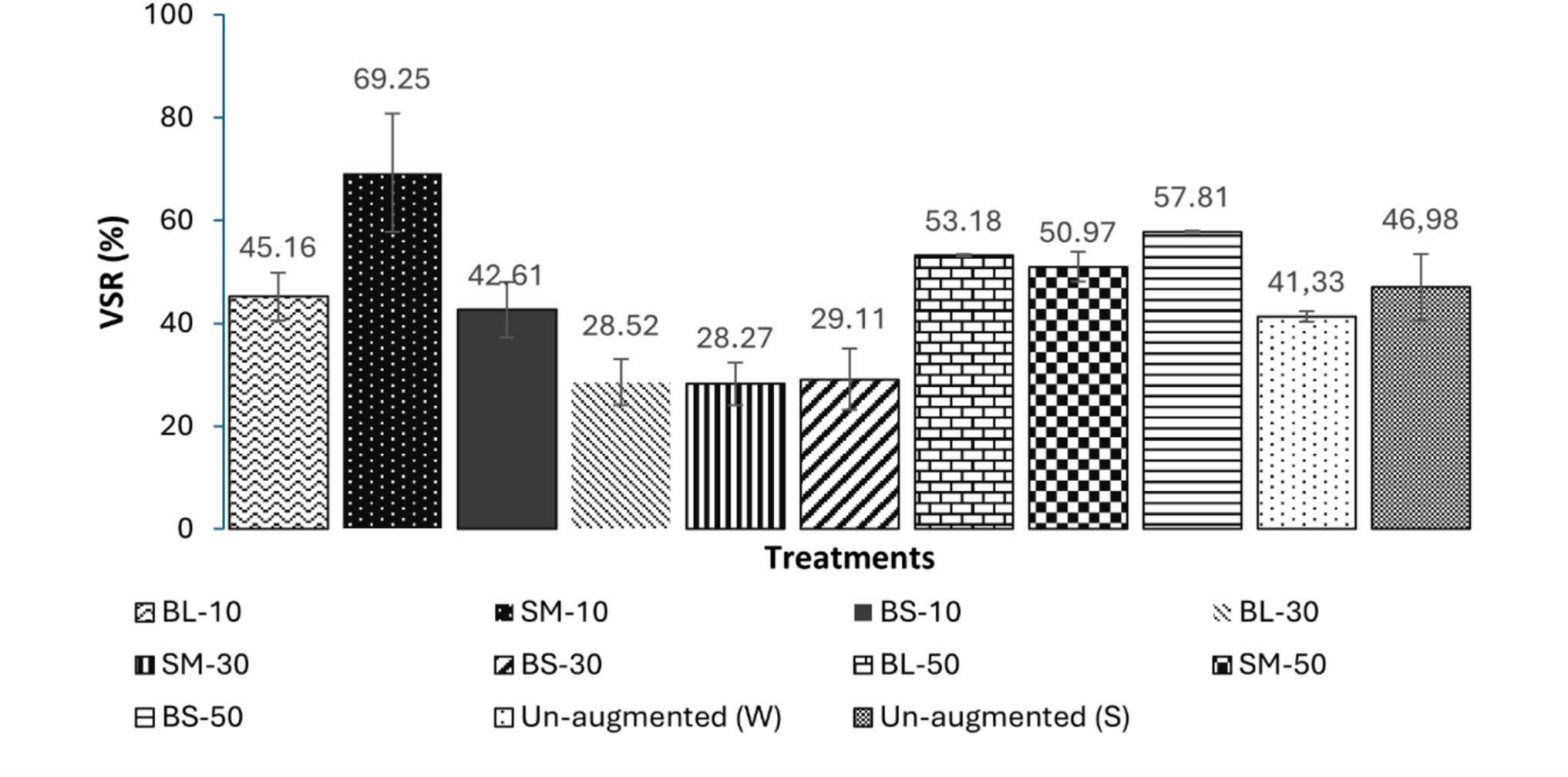
The volatile solids efficiency (VSR %) of augmentation treatments that displayed an effect on AD either through enhanced productivity or biomethane yields

Although the bioaugmentation inoculum sizes below 20 × 10^11^ CFU/mL did not increase the biomethane yield (Fig. 3), bioaugmentation at these dosages improved the efficiency of volatile solids degradation (% VSR), seen in Fig. 4. Substantial improvements in the %VSR as a result of bioaugmentation was observed for the full range of inoculum sizes investigated, with a maximum observed for SM-10 (69%), which represented an improvement of 68% in comparison to the control (Fig. 4). Such improvements in solids degradation due to bioaugmentation have been previously reported for sewage sludge (VSR improvements of 46.4–49.2%) and corn stover (64.2% improvement) [80, 81]. An improvement in % VSR without an increase in the methane yield may occur due to changes in substrate utilization rates once the microbial community is altered by augmentation. A similar contradictory trend was reported by Nabaterega et al. [82], substantiating that the relationship between VS removal and methane yield is not always linear.

#### Impact of bioaugmentation on microbial community succession

Bioaugmentation treatment SM-30 was further assessed for its impacts on microbial population dynamics and degradation of the lignocellulosic components of the substrate in the AD system, since *S. marcescens* as an augmenting agent in lignocellulosic AD is scarcely reported (e.g. Obi et al. [28, 76] on *S. marcescens* 39_H1). The alpha diversity index assesses the number of microbial species within a local, functional community. In the current study, three alpha diversity indices namely, the Shannon-, Simpson- and Observed index, were investigated and compared for treatment SM-30 and the un-augmented digester (Table 5). The Shannon index describes the number of various species present (richness), and the comparative abundance of the various species present in a system (evenness), with an increase in value over time representing a more equal abundance of each species type present Additionally, the Simpson index, which focuses on the evenness of the dominant species present, and the Observed taxa index which quantifies the total amount of different taxa, was observed [83].

**Table 5:** The three alpha diversity indices (Shannon-, Simpson- and Observed index) of the augmented (SM-30) and un-augmented AD systems

	Un-augmented (Initial)	Un-augmented (Final)	SM-30 (Initial)	SM-30 (Final)
Shannon index	2.93	2.90	3.47	4.98
Simpson index	0.85	0.75	0.91	0.98
Observed species (OTU)	855	1142	1041	1304

**Fig. 5:**
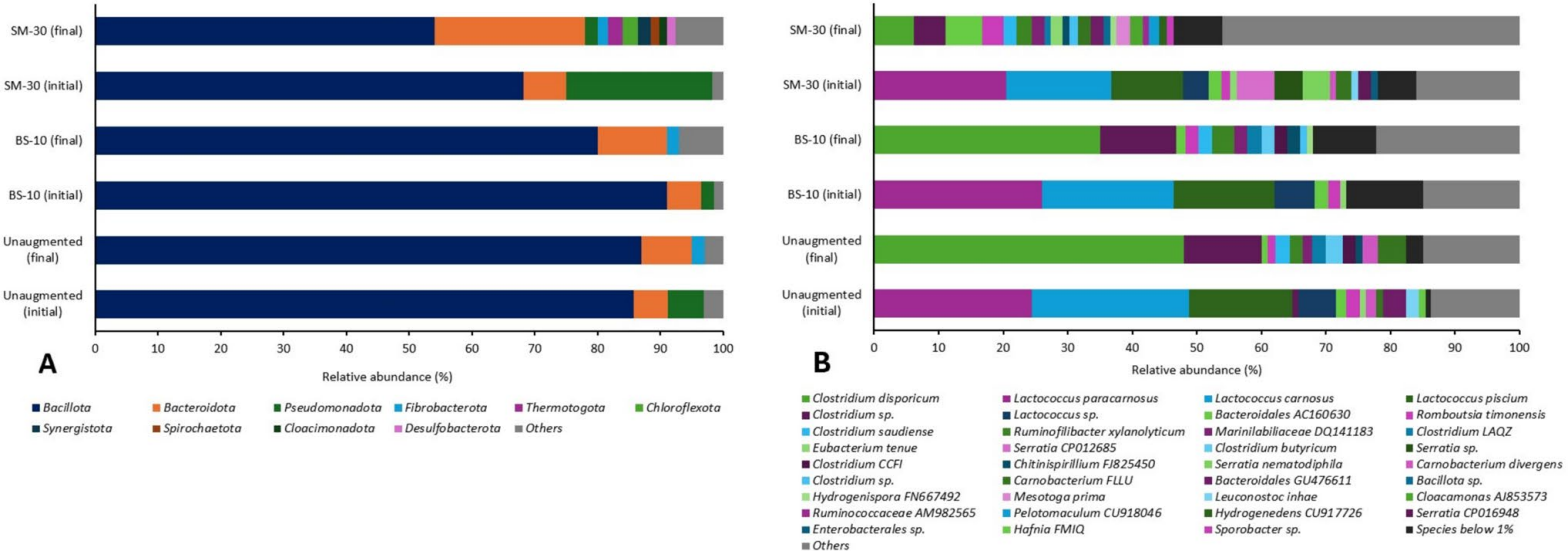
The relative abundance of the bacterial phylum (**A**) and species (**B**) found in the augmented and un-augmented systems. Phyla exhibiting a relative abundance below 1% were categorised as “others” and include *Hydrogenedentota, Thermodesulfobacteruota, Acidobacteriota, Mycoplasmatota, Afribacterota, Verrucomicrobiota, Planctomycetota, Actinomycetota, Myxococcota,* and other phyla that could not be identified. Species exhibiting a relative abundance of less than 1% are categorised as “below than 1%” and include *Brochothrix campestris, Romboutsia sp., Peptostreptococcaceae sp., Turicibacter* CP013476, *Terrisporobacter petrolearius, Sedimentibacter* F825495, *Eubacteriales sp., Aminivibrio* EU887808 and *Treponema* GU476603. Unidentified species are categorised as “others”

A collective overview of the alpha index indicated that SM-30 bioaugmentation shifted the AD microbial population towards a higher diversity, which increased its richness and evenness (Table 5). More specifically, the Shannon index radically shifted from 3.47 to 4.98 in the augmented system of SM-30, in comparison to the un-augmented system, which remained fairly stagnant between 2.90 and 2.93. The shift in the Shannon index indicated that the inoculation with *S. marcescens* promoted the abundance of taxa with a previously smaller population size, which suggested their increased functionality in the system had a contributing role in increasing the volumetric productivity within the system. The bacterial community structure of treatment SM-30 was compared against its un-augmented control at the phylum and species level, as displayed in Figs. 5.

*The Firmicutes (Bacillota*) phylum was dominant in all digesters at the end of digestion (approximately 50–80%), with identification at the species level revealing the specific dominance of *Clostridium disporicum* at a 47.76% relative abundance (RA) in the un-augmented control digestions (Fig. 5). Typically found as a pathogen in animal manure [84],* Clostridium disporicum* is known to metabolise biomass-derived sugars such as glucose, instead of the lignocellulosic biomass polymers themselves [85, 86]. This species’ dominance in the un-augmented reactor at the end of digestion thus describes the low volumetric productivity and low biomethane yield from the system, due to the rate-limited hydrolysis step for lignocellulose.

Figure 5A shows that at the end of the digestions, the abundance of the *Bacillota* phylum was reduced from 91 to 80%, and 68 to 54% when augmented with BS-10 and SM-30, respectively. Specifically, the introduction of *S. marcescens* led to an increase in the abundance of *Bacteroidota* from around 7 to 24% and an increased presence of eight more phyla. The *Spirochaetota* and *Synergistota* phyla found in the final SM-30 system have been reported to have carbohydrate-active enzyme (CAZyme) families, which are crucial to the degradation of complex carbohydrates [87]. Figure 5B also shows an increase in the relative abundance of *Ruminococcaceae* and *Clostridiaceae,* which have been reported to play a key role in lignocellulosic degradation, with cellulolytic microorganisms from *Bacteroides, Ruminiclostrdium, Enteroccous* and *Parabacteroides* genera exhibiting the capacity to encode for several cellulose and hemicellulose-degrading CAZymes when acclimatised to lignocelluloses [16].

A study by Obi et al. [28] which focused on the augmentation of AD using *S. marcescens* for the degradation of water hyacinth, described a similar digester dominance by *Firmicutes* and *Bacteroidota*. *Firmicutes* and *Bacteroidota* are often found in AD systems of various substrates, commonly lignocellulosic biomass due to their association with fermentation and carbohydrate degradation, for example, *Bacteroidota* is known to metabolise cellobiose into acetic acid [28, 85, 86]. Thus, their relatively high presence in reactor SM-30 suggests that, although the persistence and survival of *S. marcescens* was low, its introduction led to a shift in the microbial community and had an impact on enhancing this system’s lignocellulosic digestion process.

However, it should be noted that due to the limited quantification of the species at the end of SM-30 digestion (50% abundance of unassigned taxa), it is difficult to specifically discover the contributing species that were associated with the improved productivity. Based on the data available, a clear reduction in the abundance of *Lactococcus* species at the end of the SM-30 digestion was observed, which is indicative of the antagonistic action of *S. marcescens*, discussed in Sect. 3.3.1. *Lactococcus* species are not typically found in AD systems and their presence and dominance in the initial samples may be indicative of the source of the inoculum, i.e. a dairy farm.

## Conclusion

The present study demonstrated that the bioaugmentation with the cellulolytic strains *B. subtilis, B. licheniformis* and *S. marcescens* successfully improved the AD of lignocellulosic, pretreated corn stover co-digested with food waste. This was indicated by enhanced biomethane production (0.35–33.52%), increased degradation of lignocelluloses and solids, reduced digestion time by between 2 and 11 days, and improved volumetric productivity of the AD process. A minimum bioaugmentation inoculum size of 20 × 10^11^ CFU/mL was required to substantially improve the biomethane yield. At this inoculum size, augmentation with *B. subtilis* increased process performance by 33% and achieved the highest biomethane yield of 525.35 NmL/gVS at a microbial loading of 20 × 10^11^ CFU/mL. *S. marcescens* treatment (12 × 10^11^ CFU/mL) increased the bacterial α-diversity and boosted the relative abundance of pivotal lignocellulosic-degrading taxa, such as *Firmicutes* in the microbial population. Appropriate bioaugmentation is dependent on the nature of the microorganisms and can positively impact the anaerobic digestion of lignocellulosic biomass, as observed by these cellulolytic, facultative anaerobes as pure cultures.

## Supplementary Information

Below is the link to the electronic supplementary material.

Supplementary file1 (DOCX 74 KB)

## Data Availability

Additional data will be made available on request.
