# Supplementary material for: Enhancing the biomethane production from lignocellulosic residues through bioaugmentation of anaerobic digestion
Source: Bioprocess Biosyst Eng. 2025 Aug 18;48(12):1983–97. doi: 10.1007/s00449-025-03223-4 (PMC12540539; doi:10.1007/s00449-025-03223-4)
Supplement: Supplementary file 1 — Supplementary file1 (DOCX 74 KB) [file 449_2025_3223_MOESM1_ESM.docx]

**Table 1:** P values obtained from post-hoc (Fischer LSD) test comparing the methane yield achieved by each bioaugmentation treatment to its unaugmented control (treatments indicated by an asterisk (*) were compared to the winter (W) control)

| **Treatment** | **Methane yield (NmL/gVS)** | **P value** |
| --- | --- | --- |
| BL-1 | 362.62 | **0.0282** |
| BL-2 | 372.06 | 0.1225 |
| BL-3 | 367.28 | 0.0606 |
| BL-5 | 390.46 | 0.8261 |
| BL-10* | 447.72 | 0.3786 |
| BL-30* | 406.35 | 0.0737 |
| BL-50 | 449.01 | **0.0002** |
| BS-1 | 354.39 | **0.0063** |
| BS-2 | 360.23 | **0.0186** |
| BS-3 | 355.69 | **0.0080** |
| BS-5 | 391.77 | 0.9122 |
| BS-10* | 460.17 | 0.0945 |
| BS-30* | 397.46 | **0.0199** |
| BS-50 | 525.35 | **0.0000** |
| SM-1 | 350.62 | **0.0029** |
| SM-2 | 347.83 | **0.0017** |
| SM-3 | 375.20 | 0.1861 |
| SM-5 | 403.17 | 0.5260 |
| SM-10* | 415.00 | 0.2309 |
| SM-30* | 442.32 | 0.5960 |
| SM-50 | 431.82 | **0.0003** |
| Unaugmented control (S) | 393.46 | **0.0103^+^** |
| Unaugmented control (W)* | 436.12 |  |

+: unaugmented control runs conducted in summer and winter were compared to each other and were found to be statistically different

**Figure 1** Plot illustrating the optical density (at 600 nm) over time of S. marcescens, B. subtilis, and B. licheniformis grown at 37 °C, 120 rpm
